# Evaluation of neoadjuvant immunotherapy in resectable gastric/gastroesophageal junction tumors: a meta-analysis and systematic review

**DOI:** 10.3389/fimmu.2024.1339757

**Published:** 2024-01-30

**Authors:** Jincheng Wang, Ti Tong, Guangxin Zhang, Chengyan Jin, Haiping Guo, Xueying Liu, Zhengxiao Zhang, Jindong Li, Yinghao Zhao

**Affiliations:** Department of Thoracic Surgery, Second Hospital of Jilin University, Changchun, China

**Keywords:** neoadjuvant immunotherapy, efficacy, safety, meta-analysis, resectable gastric cancer/gastroesophageal junction tumors, combination therapy

## Abstract

**Background:**

Neoadjuvant therapy for resectable gastric cancer/gastroesophageal junction tumors is progressing slowly. Although immunotherapy for advanced gastric cancer/gastroesophageal junction tumors has made great progress, the efficacy and safety of neoadjuvant immunotherapy for locally resectable gastric cancer/gastroesophageal junction tumors have not been clearly demonstrated. Here, we conducted a systematic review and meta-analysis to assess the efficacy and safety of neoadjuvant immunotherapy and advance the current research.

**Methods:**

Original articles describing the safety and efficacy of neoadjuvant immunotherapy for resectable gastric cancer/gastroesophageal junction tumors published up until October 15, 2023 were retrieved from PubMed, Embase, the Cochrane Library, and other major databases. The odds ratios (OR) and 95% confidence intervals (CIs) were calculated for heterogeneity and subgroup analysis.

**Results:**

A total of 1074 patients from 33 studies were included. The effectiveness of neoadjuvant immunotherapy was mainly evaluated using pathological complete remission (PCR), major pathological remission (MPR), and tumor regression grade (TRG). Among the included patients, 1015 underwent surgical treatment and 847 achieved R0 resection. Of the patients treated with neoadjuvant immunotherapy, 24% (95% CI: 19%–28%) achieved PCR and 49% (95% CI: 38%–61%) achieved MPR. Safety was assessed by a surgical resection rate of 0.89 (95% CI: 85%–93%), incidence of ≥ 3 treatment-related adverse events (TRAEs) of 28% (95% CI: 17%–40%), and incidence of ≥ 3 immune-related adverse events (irAEs) of 19% (95% CI: 11%–27%).

**Conclusion:**

Neoadjuvant immunotherapy, especially neoadjuvant dual-immunotherapy combinations, is effective and safe for resectable gastric/gastroesophageal junction tumors in the short term. Nevertheless, further multicenter randomized trials are required to demonstrate which combination model is more beneficial.

**Systematic review registration:**

https://www.crd.york.ac.uk/PROSPERO/display_record.php?RecordID=358752, identifier CRD42022358752.

## 1 Introduction

Gastric cancer (GC), representing the fifth most common malignancy and one of the third most common cancer-related causes of death, is responsible for approximately 33% of all cancer-related deaths worldwide, and the highest mortality and incidence rates are found in East Asia (1). The junction of the esophagus and stomach (termed the gastroesophageal junction [GEJ]) is the area of transition between the esophageal squamous epithelium and the pancreatic gland columnar epithelium. Adenocarcinoma of the esophagogastric junction (AEG) is a tumor type with different biological behavior and clinical features from squamous cell carcinoma and gastric adenocarcinoma. AEG is separated into three types based on the distance from the tumor center to the GEJ – a classification first proposed by Siewert in 1999 (2–5). The majority of these tumors are histologically adenocarcinomas. The main treatment option for early and locally advanced tumors is surgical resection. Nevertheless, there is only a 10%–33% 5-year overall survival (OS) rate for patients treated with surgery alone (6–8). Therefore, it is a serious challenge to treat these patients appropriately and improve their survival rates.

In gastric and gastroesophageal junction cancers, neoadjuvant therapy is a well-established practice for reducing tumor burden, assessing tumor response preoperatively, and improving OS (9). While the landmark phase III MAGIC trial established perioperative ECF/ECX chemotherapy for resectable G/GEJ cancers as the standard of care period (10), the recently published CROSS trial established neoadjuvant radiotherapy as a valid treatment option for esophageal and GEJ tumors (7, 11, 12). To date, three completed randomized trials have directly compared neoadjuvant radiotherapy (NACRT) with neoadjuvant chemotherapy (NAC) and found that NACRT increased pathological complete remission rates and margin-negative resection rates without increasing OS (13–15). Moreover, a previous meta-analysis highlighted that the incidence of > 3 treatment-related adverse events (TRAEs) in the NAC group was as high as 25.7% in patients with resectable gastric cancer (16). It was also noted that the treatment-related complications were similar between the NAC and NACRT groups, while the postoperative complications were more severe in the NACRT group (15). As a result, there is promise that a new, more effective, neoadjuvant regimen with reduced toxicity will improve clinical outcomes in patients with G/GEJ tumors without increasing the incidence of adverse events.

As medicine continues to advance, immunotherapy has begun to gain approval in the clinical setting, thus changing the landscape of oncology treatment, with satisfactory results observed for the treatment of melanoma and non-small-cell lung cancer (17, 18). Immune checkpoint inhibitors, mainly consisting of programmed cell death protein 1 (PD-1) and programed cell death ligand 1 (PD-L1), have revolutionized the treatment of malignant tumors. PD-1 is expressed by activated lymphocytes and is expressed in combination with ligands, including PD-L1. By blocking the immune response and promoting immune escape, it further promotes the development of various malignancies and disease progression (19, 20). A recent study demonstrated that, compared to adjuvant immunotherapy, neoadjuvant immunotherapy showed significantly high therapeutic efficacy in eradicating metastases in a preclinical mouse model through systemic expansion of tumor-specific CD8^+^ T cells in peripheral blood and organs (21). Based on these findings, it is possible that neoadjuvant PD-1 blockers activate effective systemic immunity and consequently obliterate residual micrometastases after surgical resection of the primary tumor. Moreover, conventional chemotherapy has been shown to potentiate tumor antigenicity, which interferes with suppressive immune pathways and increases effector T cell responses (22). The efficacy of PD-1 receptor blockers can be enhanced by combining them with appropriate chemotherapeutic agents, especially for less immunogenic tumors with poor chemotherapy sensitivity (23, 24).

A multi-study meta-analysis will provide a more promising alternative to several neoadjuvant treatment strategies and enhance self-confidence regarding future clinical trials for neoadjuvant immunotherapy. The purpose of this meta-analysis, based on existing data, is to provide evidence for the efficacy and safety of neoadjuvant immunotherapy for resectable G/GEJ tumors and to offer options for further treatment of locally advanced G/GEJ tumors with better survival benefits in the future.

## 2 Materials and methods

This systematic review and meta-analysis was conducted and reported according to the Preferred Reporting Items for Systematic Reviews and Meta-Analyses (PRISMA) guidelines. The trial protocol can be found in PROSPERO (registration number: CRD42022358752.

### 2.1 Search strategy and study selection

Using PubMed, Embase, and the Cochrane Library, we performed a comprehensive search for articles on neoadjuvant immunotherapy for resectable G/GEJ tumors published in English up to October 15, 2023. We also conducted a retrospective search for the latest unpublished data on clinical trials of neoadjuvant immunotherapy for resectable G/GEJ tumors conducted at international oncology congresses, such as ASCO and ESMO, up to October 15, 2023. These medical databases were searched for terms such as “gastric cancer/gastroesophageal junction tumor,” “neoadjuvant therapy,” and “immunotherapy” (which includes all currently known ICIs). The full reference list of all searched texts was filtered to further identify potentially relevant studies.

### 2.2 Selection criteria and data extraction

Publications that met the following criteria were selected: 1. Publications that reported tissue-confirmed resectable G/GEJ tumors; 2. Clinical trials currently applied in clinical practice or registered; 3. Reports containing comprehensive scenarios, patient information, and a minimum critical clinical outcome with respect to PCR, MPR, TRG, TRAEs, irAEs, surgical complication rates, surgical resection rates, and operative delay rates. Publications that met the following criteria were excluded: 1. Presence of inoperable or advanced metastatic disease; 2. At no point did the study focus on MPR, PCR, TRG, TRAEs, irAEs, or surgical resection rates; 3. Enrollment of fewer than ten patients; 4. A lack of available effective data to assess the effectiveness and safety of neoadjuvant immunotherapy in combination with chemotherapy; 5. The existence of duplicate publications. Two investigators (JCW and ZXZ) independently selected a list of the retrieved publications. Following a review by a senior researcher (YHZ), any disagreements were resolved by discussion and consensus between the two reviewers, which was followed by a search of the full text to assess its eligibility. We searched and browsed each of the citations of the included studies to ensure that no relevant studies were missing.

### 2.3 Data abstraction

In the present meta-analysis, both investigators extracted the data separately. The following information was recorded for each study: first author, year of publication, clinical trial, NCT code, ICI drug, sample size, median age, MPR, PCR, TRG0-3, CR, PR, SD, ORR, DCR, TRAEs, irAEs, surgical complication rate, and surgical resection rate. In this meta-analysis, as most studies that met the inclusion criteria were conference abstracts, some epidemiological data were incomplete, such as the male to female ratio and median age. Studies for which data could not be extracted to calculate the key clinical outcomes described above from the articles, or for which data were discussed but not presented as raw data at international meetings, could not be included. Each study was reviewed several times to ensure that there were no missing or mislabeled data. Any differences regarding inclusion were addressed by discussion or by a third-party investigator who decided whether to incorporate the study. In the case of incomplete literature, the original authors were contacted for additional information wherever possible.

### 2.4 Statistical analysis

The meta-analysis was primarily undertaken using Review Manager version 5.4 (RevMan; (Cochrane Collaboration), which is a professional software provided by the Cochrane Collaboration (25). As most of the included studies were single-arm clinical trials with PCR and MPR representing the predominant outcome indicators, the research team used noncomparative binary data for meta-analysis in RevMan software. P-values and standard errors (SE(p)) were calculated by the following formula: p = ln(odds) = ln(X/(n-x)). SE(p) = SE(ln(odds)) = √1/X+1 (n-x). The dominance odds ratio (OR) and 95% confidence interval (CI) were used as efficacy evaluation indicators. For heterogeneity, the χ^2^ test and I^2^ test were used. The included studies were taken out sequentially for sensitivity analysis, which revealed that the combined results were not significantly affected by each individual trial. A random-effects model was used where heterogeneity was significant; the alternative was to use a fixed-effects model. P-values < 0.05 were considered statistically significant. Higgins I^2^ statistic < 50% was considered low heterogeneity, while > 50% of the statistic was considered high heterogeneity. Subgroup analysis was conducted to pinpoint the source of heterogeneity and factors related to clinical outcomes. RevMan 5.4 software and Stata/SE 15.0 software were used for statistical analysis of the data.

### 2.5 Assessments of publication bias and study quality

The quality of the included studies was assessed using the risk of bias assessment tools recommended by the Cochrane Handbook 5.1.0, including: (1) random assignment method; (2) allocation concealment; (3) whether participants and investigators were blinded; (4) whether efficacy was evaluated using blinded methods; (5) completeness of outcome information; (6) selective reporting of results; and (7) other biases. A qualitative assessment was independently performed by two investigators, while disagreements were decided by discussion between two or third-party investigators. The Begg test was used to test for possible publication bias in clinical studies.

## 3 Results

### 3.1 Characteristics of the included studies

**Figure 1:**
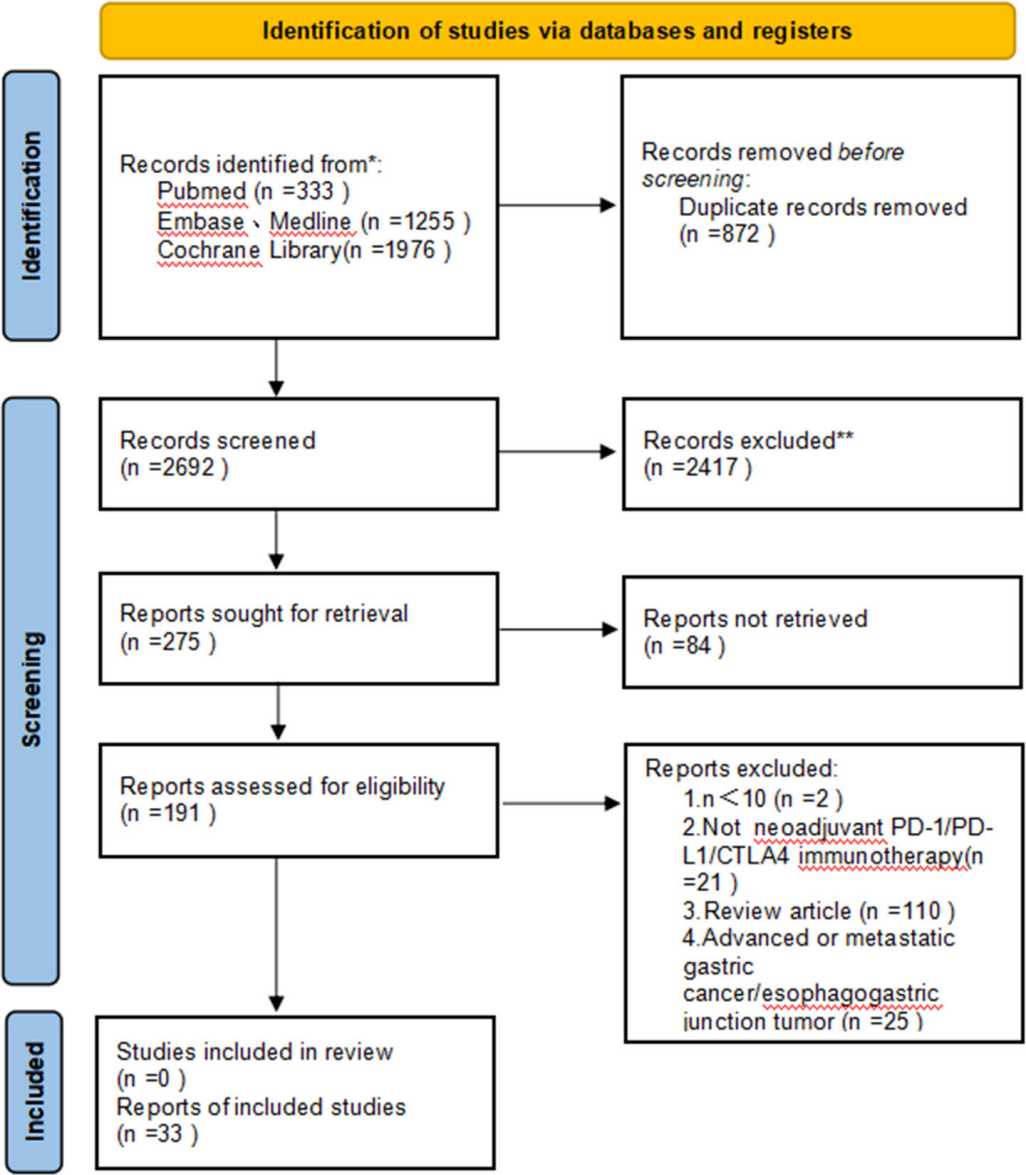
Preferred Reporting Items for Systematic Reviews and Meta-Analyses (PRISMA) diagram of the study selection.

As a result of the literature study, we identified 3,564 potentially relevant papers. After removing duplicates, there remained 2,692 papers to be analyzed. We selected 191 articles for extensive analysis by screening their titles and abstracts. After full-text screening, 33 papers fulfilled the inclusion criteria and were included in our systematic review. The main characteristics of the included studies are summarized in **Table 1**. We recorded the total number of screened, selected, and excluded studies in a prismatic flow diagram (**Figure 1**).

**Table 1:** Main characteristics of included studies.

Author year	NCT number	Study design	TYPE	PD-1/ PD-L1	Combination therapy	Clinical stage	No. of patient	Median age	Tumor type	Preoperative cycle(W)	TTS (W)	Preoperative PD-L1 CPS	dMMR/pMMR	Her2
T.Alcindor 2020 (26)	NCT03288350	nICT	Single-arm	A	mDCF	cT_3_N_any_	28	45-78	G/GEJ	4	2	–	–	–
Raufi A.G 2022 (27)	NCT02918162	nICT	Single-arm	P	CAPOX	–	34	–	G/GEJ	4	3	–	–	–
Haiping Jiang 2022 (28)	NCT04065282	nICT	Single-arm	S	CAPOX	cT_3- 4_N_any_	36	65.5	G/GEJ	3	1-4	CPS ≥1 21 CPS <1 11 Unknown 4	–	–
Deanna Huffman 2021 (29)	NCT04341857	nICT	Single-arm	S	FLOT	T_3_N_any_ or higher stage	17	–	G/GEJ	3	–	–	–	–
Ying Liu 2020 (30)	NCT03939962	nICT	Single-arm	C	FOLFOX	≥T_2_N_any_	16	57	G/GEJ	4	2-4	–	–	–
Zimin Liu 2022 (31)	ChiCTR2000030610	nICT	RCT	C	FLOT	–	33	63	G/GEJ	4	–	–	–	–
Weijing Sun 2023 (32)	NCT03488667	nICT	Single-arm	P	mFOLFOX6	T_1_N_1-3_ or T_2-3_N_any_	37	65	G/GEJ	3	4-8	–	–	–
Tao K. 2022 (33)	NCT04890392	nICT	Single-arm	T	SOX	–	21	–	G/GEJ	3	–	–	–	–
Yara L. Verschoor 2022 (34)	NCT03448835	nICT	Single-arm	A	DOC	–	20	–	G/GEJ	5	–	–	pMMR 18 dMMR 2	–
S-E. Al-Batran12021 (35)	–	nICT	RCT	A	FLOT	≥cT_2_N_any_	10	–	G/GEJ	8	–	–	–	–
Xiaohuan Tang 2022 (36)	–	nICT	Single-arm	P/N	SOX/CAPOX	cT_2-4_N_1-3_	75	64	G/GEJ	2-6	3-5	CPS >1 46 CPS≤1 12 Unknown 17	pMMR 59 dMMR 14	Positive 10
Yuping Yin 2022 (37)	NCT04890392	nICT	Single-arm	T	SOX	–	32	60.5	G/GEJ	3	4	–	–	Negative
Honghai Guo 2022 (38)	ChiCTR2000030414	nICT	Single-arm	S	CAPOX	cT_3-4_ N_any_	30	62	G	4	–	CPS< 1 11 CPS≥1 19	pMMR 13 dMMR 1	Negative
Qi Jiang 2023 (39)	NCT04890392	nICT	RCT	T	SOX/FOLFOX	cT_3-4a_N_any_ /cT_1-4a_N_+_	50	–	G	2-4	–	–	–	Negative
Ju-Li Lin 2022 (40)	–	nICT	RCT	C	S-1+Nab-PTX	cT_4_N_1-3_	33	61.9	G	3-4	–	–	–	–
Jia-lin Tang 2022 (41)	–	nICT	Single-arm	L	FP	cT_2-4a_N_any_	30	64.5	G/GEJ	3	4-6	–	pMMR 26 Unknown 4	–
Xuchen Zhang 2023 (33)	QYFYWZLL27406	nICT	RCT	C/S/T/N	FLOT/SOX	T_3-4a_N_0-3_	34	60.1	G	2-4	4	–	pMMR 29 dMMR 5	–
Ding X 2022 (42)	–	nICT	Single-arm-	S	SOX	II-IV_A_	21	56	G/GEJ	3	–	–	–	–
Jiang Z 2022 (43)	NCT04119622	nICT	Single-arm	T	CAPOX	cT_3-4_ N_any_ /T_1-2_ N_2-3_	27	–	G/GEJ	6	–	–	–	–
Xue Wang 2023 (44)	–	nICT	Single-arm	C/S/T	FLOT/CAPOX	T_3-4a_N_0-3_	42	58	G	2-4	–	CPS<1 14 CPS≥1 24 Unknown 4	pMMR 38 dMMR 4	Negative 37 Positive 5
M. Alsina 2023 (45)	NCT03979131	nICT	Single-arm	A	FLOT	≥cT_2_ N_+_	40	–	G/GEJ	4	–	–	–	–
Kim H.-D2023 (46)	4221555	nICT	Single-arm	D	DOS	cT_2/3_N_+_ /cT_4_N_any_	35	–	G	3	–	–	pMMR 35	–
Hirotaka Hasegawa 2022 (47)	JapicCTI-183895	nI	Single-arm	N	–	T_2-4a_N_0-2_	31	69	G	2	–	CPS<1 11 ≥1to<10 11 ≥10 9	pMMR 24 dMMR 7	–
Thierry André 2023 (48)	NCT04006262	nII	Single-arm	N	Ipilimumab	cT_2-3_N_0-1_	32	65.5	G/GEJ	2-6	4-6	–	–	–
Pietrantonio F 2023 (49)	NCT04817826	nII	Single-arm	D	Tremelimumab	cT_2-4_N_any_	15	–	G/GEJ	3	–	–	–	–
Mojun Zhu 2023 (50)	NCT02730546	nICRT	Single-arm	P	CROSS	cT_1-3_N_any_	31	62	G/GEJ	–	–	CPS <1 11 1–9 11 ≥10 8 Unknown 1	pMMR 21 dMMR 10	–
Jia Wei 2023 (51)	ChiCTR1900024428	nICRT	Single-arm	S	S-1+Nab-PTX+chemoradiotherapy	T_3-4b_N_1-3_	34	65.5	G/GEJ	7	1-3	CPS<1 16 ≥1and<5 5 ≥5 11 Unknown 2	pMMR 33 dMMR 1	HER2status 0 19 1+ 10 2+ 5
Zhaoqing Tang 2022 (52)	NCT03631615	nICRT	Single-arm	C	CAPOX+chemoradiotherapy	T_3-4a_N_+_	36	65.5	G/GEJ	5	–	CPS<1 16 CPS≥1 9	pMMR 33	–
Chao Xu 2022 (53)	–	nICT+apatinib	Single-arm	PD-1	SOX+ apatinib	T_3-4a_N_1 -3_	30	58.2	G	3	3-5	–	–	–
Hui Xiong 2023 (54)	–	nICT+apatinib	RCT	S/C	SOX/CAPOX+ apatinib	cT_3-4a_N_1-3_	56	58.2	G/GEJ	4	–	–	–	–
SongLi 2023 (55)	NCT03878472	nICT+apatinib	Single-arm	C	SOX+ apatinib	cT_4a/b_N_+_	25	63	G	2.6	–	–	pMMR 21 dMMR 4	–
Chunjing Wang 2023 (56)	–	nICT+apatinib	RCT	S/C/T	SOX/CAPOX+ apatinib	cT_3-4a_N_1 -3_	39	57.9	G	3	–	CPS1–4 9 CPS5–9 16 CPS≥10 14	pMMR 36 dMMR 3	–
H.Zhou 2023 (57)	–	nICT+apatinib	Single-arm	S	FLOT+ apatinib	cT_3-4_	44	56.5	G/GEJ	4	–	–	–	–

nICT, Neoadjuvant immunotherapy combined with chemotherapy; nI, Neoadjuvant monoimmunotherapy; nICRT, Neoadjuvant immunotherapy combined with chemotherapy and radiotherapy; A, Avelumab; C, Camrelizumab; P, Pembrolizumab; S, Sintilimab; T, Tislelizumab; N, Nivolumab; L, LP002; D, Durvalumab; mDCF, docetaxel+cisplatin+5-fluorouracil; FLOT, docetaxel+oxaliplatin+leucovorin+fluorouracil; FOLFOX, Oxaliplatin+5-fluorouracil+leucovorin; CAPOX, capecitabine +oxaliplatin; SOX, S-1+Oxaliplatin; DOC, Docetaxel + oxaliplatin + capecitabine; FP, Cisplatin + 5-fluorouracil; CROSS, Carboplatin + paclitaxel + 41.4Gy radiation therapy; CPS, Combination positive score; dMMR, Mismatch Repair Deficiency; pMMR, Proficient mismatch repair.

Among the 33 included studies, there were seven randomized controlled trials (RCTs) (31, 35, 39, 40, 54, 56, 58) and 26 single-arm open-label cohort studies (27, 28, 30, 32–34, 36–38, 41–53, 55, 57, 59, 60). The main neoadjuvant immunotherapy drugs used were avelumab, camrelizumab, pembrolizumab, sintilimab, tislelizumab, nivolumab, LP002, and durvalumab. According to the different treatment protocols, the five treatment modalities were classified as follows: neoadjuvant monoimmunotherapy (nI), neoadjuvant dual immunotherapy (nII), neoadjuvant immunotherapy in combination with chemotherapy (nICT), neoadjuvant immunotherapy along with chemoradiation (nICRT), and neoadjuvant immunotherapy along with apatinib and chemotherapy (nAICT+apatinib). The included studies were found to have a low risk of summary bias, as shown in **Supplementary Figure 1**. A total of 1,074 patients were enrolled in 33 studies, most of whom received 2–4 cycles of neoadjuvant immunotherapy. A total of 1,015 patients underwent surgery after neoadjuvant therapy, among whom, 847 achieved R0 resection in the published results. A further detailed summary of the patient characteristics is provided in **Table 2**.

**Table 2:** Main characteristics of included studies.

Author year	pCR	MPR	TRG0	TRG1	TRG2	TRG3	R0 resection rate	Surgical resection rate	CR	PR	SD	ORR	DCR	≥3 TRAEs	≥3 irAE
T.Alcindor 2020 (26)	6/27		3/27	3/27			26/27	27/28	6/34						
Raufi A.G 2022 (27)	7/34							29/34						18/35	10/34
Haiping Jiang 2022 (28)	7/36	17/36		17/36	15/36	4/36	35/36	36/36						10/36	
Deanna Huffman 2021 (29)	2/9	6/9						9/17		6/17	11/17	9/17	17/17		
Ying Liu 2020 (30)	1/13			3/13			13/15	15/16							
Zimin Liu 2022 (31)	3/26		4/26	1/26	14/26	7/26	26/26	31/33							
Weijing Sun 2023 (32)	6/29						29/29	29/37							
Tao K. 2022 (33)	5/21	13/21						21/21						1/21	
Yara L. Verschoor 2022 (34)	9/20	14/20						20/20							2/20
S-E. Al-Batran1 2021 (35)	5/10			8/10	1/10			10/10							
Xiaohuan Tang 2022 (36)	21/75		21/75	13/75	28/75	13/75	74/75	75/75							
Yuping Yin 2022 (37)	8/32	17/32		9/32	4/32	9/32	30/30	30/30		13/32	12/32			4/32	
Honghai Guo 2022 (38)	10/30	19/30	10/30	9/30	6/30	5/30	30/30	30/30	1/30	19/30	9/30	21/30	30/30		
Qi Jiang 2023 (39)	13/50		14/50	8/50	11/50	17/50	50/50	50/50							
Ju-Li Lin 2022 (40)	7/33			13/33			32/33	33/33							
Jia-lin Tang 2022 (41)	1/30			1/27	2/27	3/27	24/30	27/30						11/30	
Xuchen Zhang 2023 (58)	8/34	13/34		13/34	12/34	9/34	33/34	34/34		26/34	8/34	26/34		10/34	
Ding X 2022 (42)	7/21						21/21	21/21						2/21	
Jiang Z 2022 (43)	3/27	4/27					26/27	27/35						4/27	1/27
Xue Wang 2023 (44)	11/42	18/42		18/42	14/42	10/42	38/44	44/44		30/42	12/42	30/42	42/42	8/42	
M. Alsina 2023 (45)	8/38						27/32	32/40							11/40
Kim H.-D 2023 (46)	9/31							31/35							
Hirotaka Hasegawa 2022 (47)	1/31	5/31					27/31	30/31						1/31	7/31
Thierry André 2023 (48)	17/29						29/29	29/32	5/32	12/32	11/32			6/32	
Pietrantonio F 2023 (49)	9/15	12/15						14/15							3/18
Mojun Zhu 2023 (50)	7/31						28/31	29/31						17/31	5/31
Jia Wei 2023 (51)	13/34	27/34					34/34	34/34						17/34	11/34
Zhaoqing Tang 2022 (52)	12/36	16/36					33/36	36/36						27/36	
Chao Xu 2022 (53)	6/30						28/30	30/30	2/30	18/30	10/30	20/30	30/30		
Hui Xiong 2023 (54)	15/56						53/56	56/56	3/56	38/56	15/56	41/56	56/56		
SongLi 2023 (55)	3/19	5/19		5/19	4/19	10/19	19/23	23/23							
Chunjing Wang 2023 (56)	9/39						38/39	39/39	2/39	27/39	10/39	29/39	39/39		
H.Zhou 2023 (57)	14/44	23/44					44/44	44/44	13/44	21/44	10/44	34/44	44/44		

PCR, complete pathological response; MPR, major pathological response; TRG, Tumor regression grade; CR, Complete response; PR, Partial response; SD, Stable disease; ORR, Objective response rate; DCR, Disease control rate; TRAEs, Treatment-related adverse event; irAE, Immune-related adverse event.

### 3.2 Quality assessment of the studies

To assess the efficacy and safety of neoadjuvant immunotherapy in 33 clinical studies, we performed a Begg test using Stata software to identify possible publication bias. The results revealed no significant publication bias because most of the data were from uncontrolled cohort clinical trials and the graphs showed a symmetrical distribution (**Supplementary Figure 2**).

## 4 Results

### 4.1 Efficacy

#### 4.1.1 Pathological complete responses

**Figure 2:**
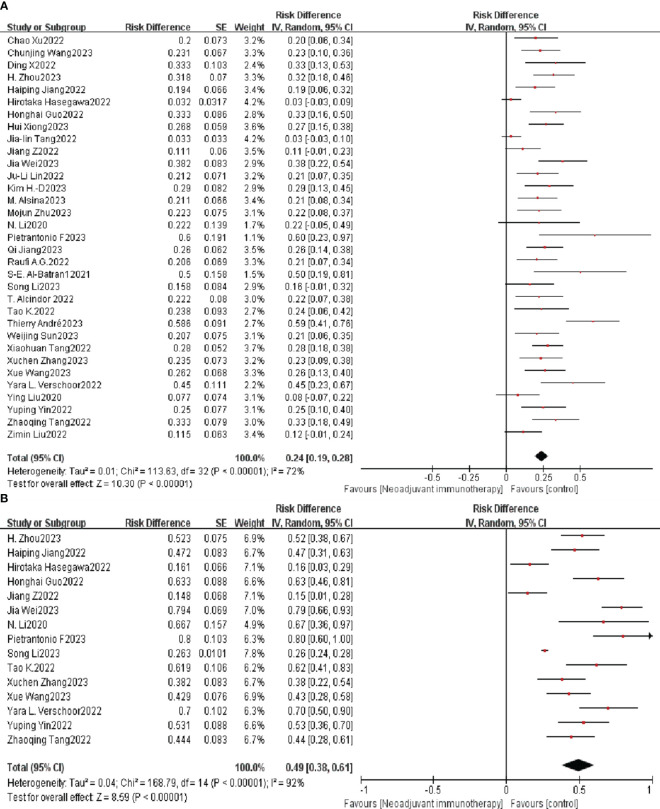
Neoadjuvant immunotherapy efficacy forest plot. **(A)** PCR and **(B)** MPR. PCR, pathological complete remission; MPR, major pathological remission.

The aggregated PCR data for 1,074 patients in 33 studies was 24% (95% CI: 19%–28%), with potential heterogeneity (I^2 =^ 72%, *p* < 0.0001) (**Figure 2A**); therefore, a random-effects model was used. Each individual OR of the eligible studies supported the effectiveness of neoadjuvant immunotherapy for resectable G/GEJ (individual OR < 1.0).

#### 4.1.2 Major pathological responses

All 15 trials had a single OR in favor of neoadjuvant immunotherapy (single OR < 1.0). Combining these 15 studies, the aggregated MPR showed a statistically significant 49% difference (95% CI: 38%–61%; *p* < 0.0001; **Figure 2B**). Because of the same significant heterogeneity (*p* < 0.0001, I^2 =^ 92%), we used a random-effects model.

#### 4.1.3 Tumor regression grade

**Figure 3:**
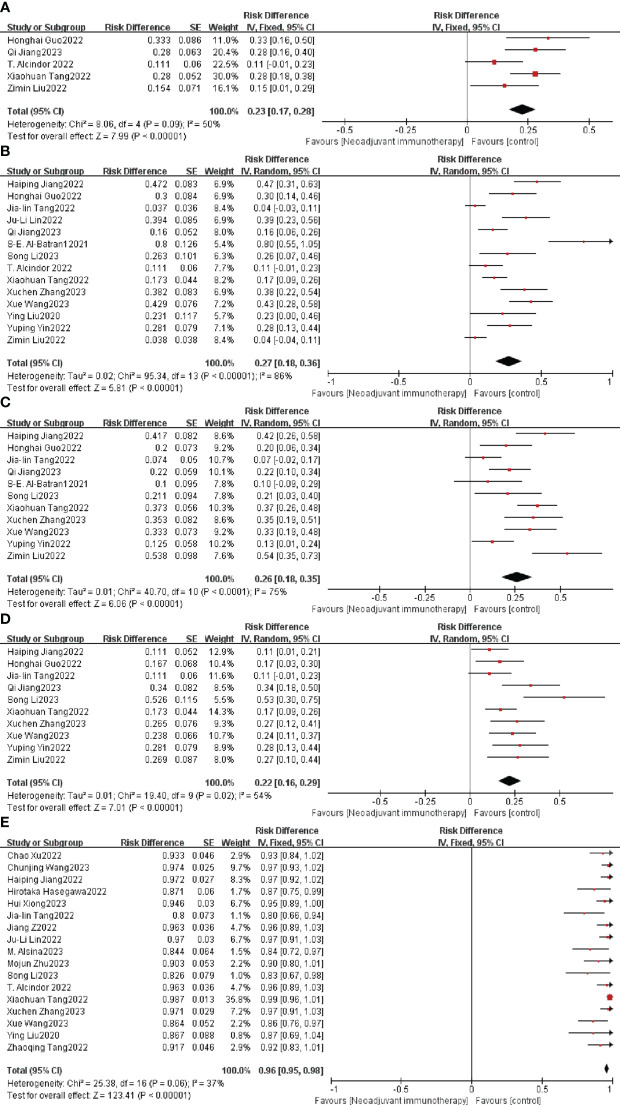
Neoadjuvant immunotherapy efficacy forest plot. **(A)** TRG0, **(B)** TRG1, **(C)** TRG2, **(D)** TRG3, and **(E)** R0 resection rate. TRG, tumor regression grade.

TRG systems that aim to categorize the amount of regressive changes after cytotoxic treatment mostly refer to the amount of therapy-induced fibrosis in relation to the residual tumor or the estimated percentage of residual tumor in relation to the previous tumor site. The combined TRG0 was 23% (95% CI: 17%–28%, I^2 =^ 50%, P = 0.09, **Figure 3A**), the merged TRG1 was 27% (95% CI: 18%–36%, I^2 =^ 86%, *p* < 0.0001, **Figure 3B**), the united TRG2 was 26% (95% CI: 18%–35%, I^2 =^ 75%, *p* < 0.0001, **Figure 3C**), and the combined TRG3 was 22% (95% CI: 16%–29%, I^2 =^ 54%, P = 0.02, **Figure 3D**), and fixed/random-effects models were used according to different I^2^ value.

#### 4.1.4 R0 Resection rate

The R0 resection rate is another important index for estimating the efficiency of neoadjuvant therapy. Nine of the 26 studies achieved a 100% R0 resection rate, and the remaining 17 studies had an individual OR < 1, with a combined OR of 96% (95% CI: 95%–98%, *p* < 0.0001; **Figure 3E**). A fixed-effects model was used given that the heterogeneity (P = 0.06, I^2 =^ 37%) was not significant.

### 4.2 Safety

#### 4.2.1 Incidence of grade ≥ 3 TRAEs

**Figure 4:**
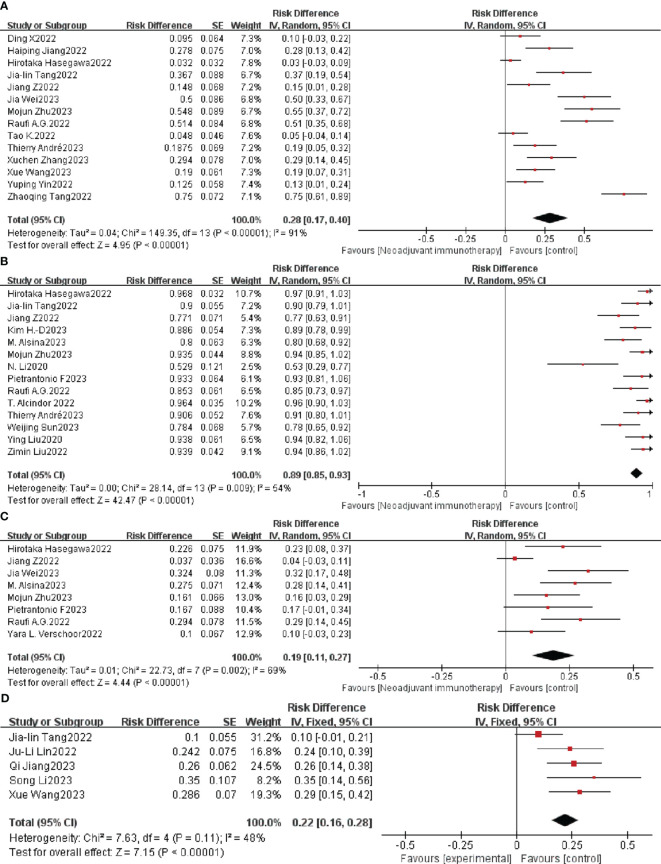
Neoadjuvant immunotherapy safety forest plot. **(A)** ≥ 3TRAEs. **(B)** Surgical resection rate. **(C)** ≥ 3irAEs. **(D)** Postoperative complication. TRAEs, treatment-related adverse events; irAEs, immune-related adverse events.

Adverse events caused by ICIs are defined as TRAEs and are evaluated by the National Cancer Institute Common Terminology Criteria for Adverse Events (NCI-CTCAE) version 5 (61), which is the key metric for evaluating neoadjuvant immunotherapy safety. A total of 117 cases of grade ≥ 3 TRAEs were reported in 14 clinical studies. Neoadjuvant immunotherapy was supported when the overall individual OR and combined analysis showed an individual OR < 1 and a combined OR of 28% (95% CI: 17%–40%); the difference was statistically significant (*p* < 0.0001; **Figure 4A**). A random-effects model was used due to the significant heterogeneity (*p* < 0.0001, I^2 =^ 91%).

#### 4.2.2 Surgical resection rate

As a percentage of patients with resected tumors versus those awaiting resection, the surgical resection rate is also an important indicator of the safety of neoadjuvant immunotherapy. Nineteen of the 33 studies had a 100% surgical resection rate. The combined OR of neoadjuvant immunotherapy was 89% (95% CI: 85%–93%; *p* < 0.0001) using a random-effects model (P = 0.009, I^2 =^ 54%; **Figure 4B**).

#### 4.2.3 Incidence of grade ≥ 3 irAEs

Adverse events associated with treatment with immune checkpoint inhibitors (ICIs) were defined as irAEs. A total of 50 cases of grade ≥ 3 irAEs were reported in eight clinical studies. Neoadjuvant immunotherapy was supported when the combined OR of grade ≥ 3 irAE with resectable G/GEJ was 19% (95% CI: 11%–27%); the difference was statistically significant (P = 0.002; **Figure 4C**). A random-effects model was used due to the significant heterogeneity (*p* < 0.0001, I^2 =^ 69%).

#### 4.2.4 Postoperative complications

Postoperative complications are other important indicators of the safety of neoadjuvant immunotherapy. Five of 33 studies mentioned postoperative complications. The combined OR was 22% (95% CI: 16%– 28%; *p* < 0.0001) using a fixed-effects model (P = 0.11, I^2 =^ 48%; **Figure 4D**).

#### 4.2.5 Drug toxicity

Drug toxicity is the other important factor used to evaluate the safety of neoadjuvant immunotherapy. In the current analysis, two (26, 32) studies mentioned grade G3/4 toxicity, including stomatitis, nausea, vomiting, diarrhea, hypothyroidism, arthralgia, neutropenia, and pneumonia, but no instances of postponement of surgery or death due to drug toxicity.

### 4.3 Sensitivity analysis

The criteria for re-examining the search, selection, and merging of studies did not reduce the heterogeneity. A sensitivity analysis was performed by removing the included studies in order, with the aim to confirm that the conjunction results were not significantly impacted by an individual trial. Of the 33 studies analyzing individual studies of PCR incidence, the two studies by Hirotaka Hasegawa (47) and Jia-lin Tang (41) contributed the most to the heterogeneity, although the weights given in these two studies were not the greatest. Heterogeneity was significantly reduced after excluding the two studies (P = 0.02; I^2 =^ 39%), and the remaining 31 combined trials still significantly demonstrated the safety of neoadjuvant immunotherapy in combination with chemotherapy (OR = 25%; 95% CI: 22%–27; *p* < 0.0001; **Supplementary Figure 3**). The most significant cause of heterogeneity in TRG3 surgical resection rates was the study by Song Li et al. (55). After removing this study, the OR for combined TRG3 in the remaining ten trials was 19 (95% CI: 15%–23%; I^2 =^ 29%, *p* < 0.0001; **Supplementary Figure 4**). The OR for the surgical resection rate in the other 14 trials combined was 92 (95% CI: 89%–94%; I^2 =^ 33%, *p* < 0.0001; **Supplementary Figure 5**). Moreover, Jiang Z’s (43) study was the main reason for the heterogeneity of irAEs. The remaining eight trials combined with an OR of 21% (95% CI: 16%–27%; I^2 =^ 20%; **Supplementary Figure 6**) remained supportive of neoadjuvant immunotherapy after exclusion (P = 0.27). In conclusion, sensitivity analyses of the study results continue to confirm the efficacy and safety of neoadjuvant immunotherapy along with chemotherapy.

### 44 Exploratory subgroup analysis

In the subgroup analysis, randomized clustering was applied to investigate potential branches of heterogeneity. We performed subgroup analysis to identify possible associations between neoadjuvant immunotherapy and different treatment regimens. The study was divided into nI, nII, nICT, nICRT and nAICT+apatinib groups. Heterogeneity was not significantly reduced because of the exclusion of MPRs as well as grade ≥ 3 TRAEs from the literature on a case-by-case basis in the sensitivity analysis. Because PCR is an important indicator of our concern, we performed subgroup analyses for all three indicators.

After subgroup analysis, the nII group and OR were much higher on PCR (OR = 59%, 95% CI: 43%–75%; I^2^ = 0%; **Supplementary Figure 7**) and MPR (OR = 80%, 95% CI: 60%–100%; **Supplementary Figure 8**), and lower than the other groups on ≥ 3TRAEs (OR = 19%, 95% CI: 5%–32%; **Supplementary Figure 9**). In contrast, the nICRT group had a much higher combined OR at ≥ 3 TRAEs (OR = 61%, 95% CI: 45%–76%; I^2 =^ 66%) than the other groups, suggesting that the short-term survival outcome of patients is influenced by the neoadjuvant immunotherapy regimen. However, from the above analysis, the efficacy and safety of neoadjuvant immunotherapy for resectable G/GEJ remains reliable.

## 5 Discussion

Regional variations exist for treating locally progressive G/GEJ cancers. In Europe, the emphasis is on perioperative chemotherapy, whereas in North America, simultaneous postoperative radiotherapy is advocated, and in Asia, D2 curative surgery in conjunction with complementary postoperative chemotherapy is favored, with surgery continuing to be a definitive treatment (62). The standard treatments for locally progressive G/GEJ tumors are both NAC and NACRT. PCR rates are high with nCRT, but it is unclear whether such high PCR rates translate into long-term survival benefits. For the first-line treatment of advanced gastric cancer, immunotherapy has been broadly applied and extensively studied in the circumoperative phase. It is possible that compound chemotherapy and immunotherapy have concerted effects and produce better antitumor effects.

At present, there remains controversy regarding the effectiveness and safety of neoadjuvant immunotherapy for resectable G/GEJ tumors; however, our study strongly supports the effectiveness and safety of neoadjuvant immunotherapy for resectable G/GEJ tumors. More importantly, not only is PCR a potent marker of excellent performance of neoadjuvant therapy in the short term but also a relatively accurate predictor with respect to gastric cancer recurrence, metastasis, and patient survival following treatment with neoadjuvant therapy (63). In our meta-analysis, the mean PCR for neoadjuvant immunotherapy was 26%, which was slightly higher than 23% in the CROSS study (11). Nine of the 33 studies had PCRs > 30%, the highest of which was the study by Pietrantonio F (49). and colleagues, with a PCR of 60%. Surprisingly, the mean MPR was 50.4% in the 15 included clinical studies, with the highest being the clinical trial by Pietrantonio F. et al. (49), with an MPR of 80%. Six studies had MPRs > 60%. PD-L1 expression is a potential biomarker for anti-PD -1/PD-L1 therapy. However, its predictive value in GC is unclear. Furthermore, it was found that microsatellite instability (MSI)/mismatch repair (MMR) levels and tumor mutation burden (TMB) may be effective markers for screening patients for potential benefit from immunotherapy (64, 65). Ten (28, 35, 36, 38, 44, 47, 50–52, 58) of the 33 included studies were found to indicate a higher incidence of PCR with MPR after neoadjuvant immunotherapy in patients with detectable CPS ≥ 1 or dMMR than in patients with CPS < 1 or pMMR. This result indicates that CPS ≥ 1 or dMMR may serve as a biomarker of prognosis in patients with resectable G/GEJ. With these promising and encouraging outcomes, we have provided abundant evidence to support the effectiveness of neoadjuvant immunotherapy.

With regard to the safety of surgery, an average R0 resection rate of 94.5% was achieved with the combination of neoadjuvant immunotherapy and chemotherapy, much higher than the 82%–85% observed in the CROSS study (11) and 84% in the FLOT4-AIO study (66). Again, this suggests a promising and attractive effect of neoadjuvant immunotherapy. Nevertheless, it is difficult to elucidate the benefit of neoadjuvant immunotherapy in prolonging survival because of the short follow-up period and the fact that complete survival data based on RCTs have not been published. However, there is promise that future OS and DFS/PFS data will provide insight into the impact and benefit of the combination of neoadjuvant immunotherapy with chemotherapy on survival over the long term.

In particular, the results of the safety analysis suggest that it is possible to proceed with neoadjuvant immunotherapy assertively. The average incidence of grade ≥ 3 TRAEs in our meta-analysis was 29.1%, showing good tolerability. As seen in the subgroup analysis, the nICRT group greatly increased the incidence of grade ≥ 3 TRAEs. The majority of ICIs have also been previously assessed in full initial clinical studies and have been used globally for the treatment of extensive advanced tumors and consequently carry significant insight into the recognition and care of undesired events, providing further evidence that TRAE is an effective treatment. In terms of surgical resection rates, neoadjuvant immunotherapy averaged 94.3%. Further, the mean incidence of grade ≥ 3 irAEs was only 19.8%, again indicating good tolerability. Five studies in the included literature mentioned postoperative complications, with 13 cases of grade ≥ 3, including two anastomotic fistulas (47), six infections, and five cardiac causes (50). Considering these consequences together, the safety of neoadjuvant immunotherapy is deemed acceptable. In the nII group, neoadjuvant immunotherapy was associated with slightly lower grade ≥ 3 TRAEs as well as grade ≥ 3 irAEs, which may indicate a synergistic effect of neoadjuvant immunization along with immunotherapy, which can achieve higher PCR and MPR and does not enhance the incidence of AEs. Of course, further clinical studies are needed to verify the feasibility of this regimen (67). ICI subgroups based on ICI type could not be analyzed because the choice of chemotherapy regimen was not identical in any of the included studies, and there was no evidence that different ICIs contributed differently to the efficacy and safety of neoadjuvant immunotherapy. Therefore, ICIs are not currently the preferred choice for neoadjuvant immunotherapy; instead, neoadjuvant immunotherapy drug selection is dependent on the individual patient and the clinical situation. Of course, additional clinical trial data are needed to support this conclusion.

This meta-analysis has several limitations. First, the majority of the included clinical studies have not reached the terminal point. Therefore, there are few clinical studies without a comprehensive regimen and available data. Additionally, as most data came from conference abstracts, these studies were not formally published in these cases, which may affect the assessment of bias and influence publication bias. However, publication bias due to article type is acceptable because leakage plots for assessing publication bias are distributed symmetrically. In addition to the metrics noted in this paper, other metrics could be used to estimate efficacy and safety, such as CR, PR, DCR, SD, PFS, OS, and time to surgery. However, we could not use these metrics because of the lack of relevant data. Another major limitation is that some trials have small sample sizes and there are so few RCTs, for which biases may result. As a result, there is a need for larger sample sizes and more RCTs for further validation in polycenter studies.

Despite the excellent results in terms of removal rates, some concerns remain. First, regarding the frequency of cycles of neoadjuvant therapy, it is unclear whether an increase in the number of cycles will improve treatment efficacy, produce better MPR rates and PCR rates, and reduce both toxicity and side effects. Second, it remains to be determined whether the sequencing of chemotherapeutic agents with immune reagents will do a better job of enhancing metrics such as PCR rates. Third, future studies are required to establish which combination of immune drugs and which treatment modality achieves maximal PCR/MPR. To conclude, a high postoperative PCR rate does not directly imply a high survival rate. However, as most of the included studies had short follow-up periods, conclusive results could not be obtained.

To summarize, our meta-analysis of the effectiveness and safety of neoadjuvant immunotherapy for resectable G/GEJ tumors suggests that the extensive use of neoadjuvant immunotherapy is clinically supported. However, because most clinical trials have not yet met their endpoints, it is important to examine the long-term outcomes and toxicity to confirm this conclusion. It is comforting that more neoadjuvant immunotherapy studies are underway, which may confirm the above conclusions in the future.

## 6 Conclusion

Neoadjuvant immunotherapy, especially neoadjuvant dual-immunotherapy combinations, is effective and safe for resectable gastric/gastroesophageal junction tumors in the short term. Nevertheless, further multicenter randomized trials are required to demonstrate which combination model is more beneficial.

## Data availability statement

The original contributions presented in the study are included in the article/**Supplementary Material**. Further inquiries can be directed to the corresponding author.

## Author contributions

JW: Conceptualization, Data curation, Formal analysis, Investigation, Methodology, Software, Writing – original draft, Writing – review & editing. TT: Conceptualization, Methodology, Writing – review & editing. GZ: Formal analysis, Software, Writing – review & editing. CJ: Formal analysis, Software, Writing – review & editing. HG: Writing – original draft, Writing – review & editing. XL: Writing – original draft, Writing – review & editing. ZZ: Data curation, Investigation, Writing – review & editing. JL: Funding acquisition, Project administration, Supervision, Visualization, Writing – review & editing. ZY: Conceptualization, Funding acquisition, Methodology, Project administration, Supervision, Visualization, Writing – review & editing.

## Supplementary material

Supplementary Figure 1 Assessment of publication bias and study quality.

Supplementary Figure 2 Publication bias test (PCR).

Supplementary Figure 3 Sensitivity analysis of the incidence of PCR.

Supplementary Figure 4 Sensitivity analysis of the incidence of TRG3.

Supplementary Figure 5 Sensitivity analysis of the surgical resection rate.

Supplementary Figure 6 Sensitivity analysis of the incidence of ≥ 3irAEs.

Supplementary Figure 7 Subgroup analysis of PCR.

Supplementary Figure 8 Subgroup analysis of MPR.

Supplementary Figure 9 Subgroup analysis of the incidence of ≥ 3TRAEs.
