# Supplementary figures and images for: Evaluation of neoadjuvant immunotherapy in resectable gastric/gastroesophageal junction tumors: a meta-analysis and systematic review
Source: Front Immunol. 2024 Jan 30;15:1339757. doi: 10.3389/fimmu.2024.1339757 (PMC10861722; doi:10.3389/fimmu.2024.1339757)

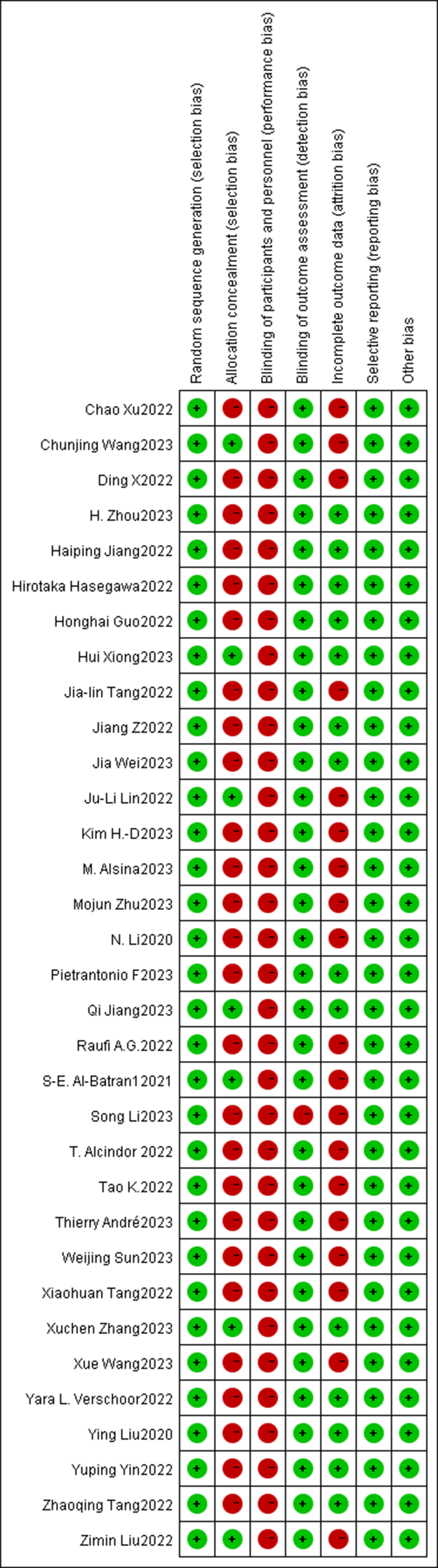

Supplement: Supplementary Figure 1 — Assessment of publication bias and study quality. [file Image_1.tif]

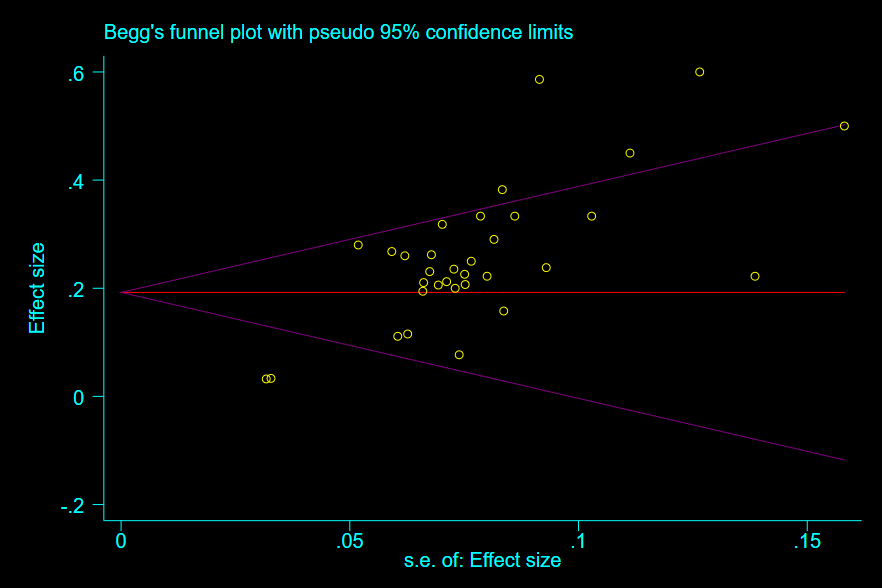

Supplement: Supplementary Figure 2 — Publication bias test (PCR). [file Image_2.tif]

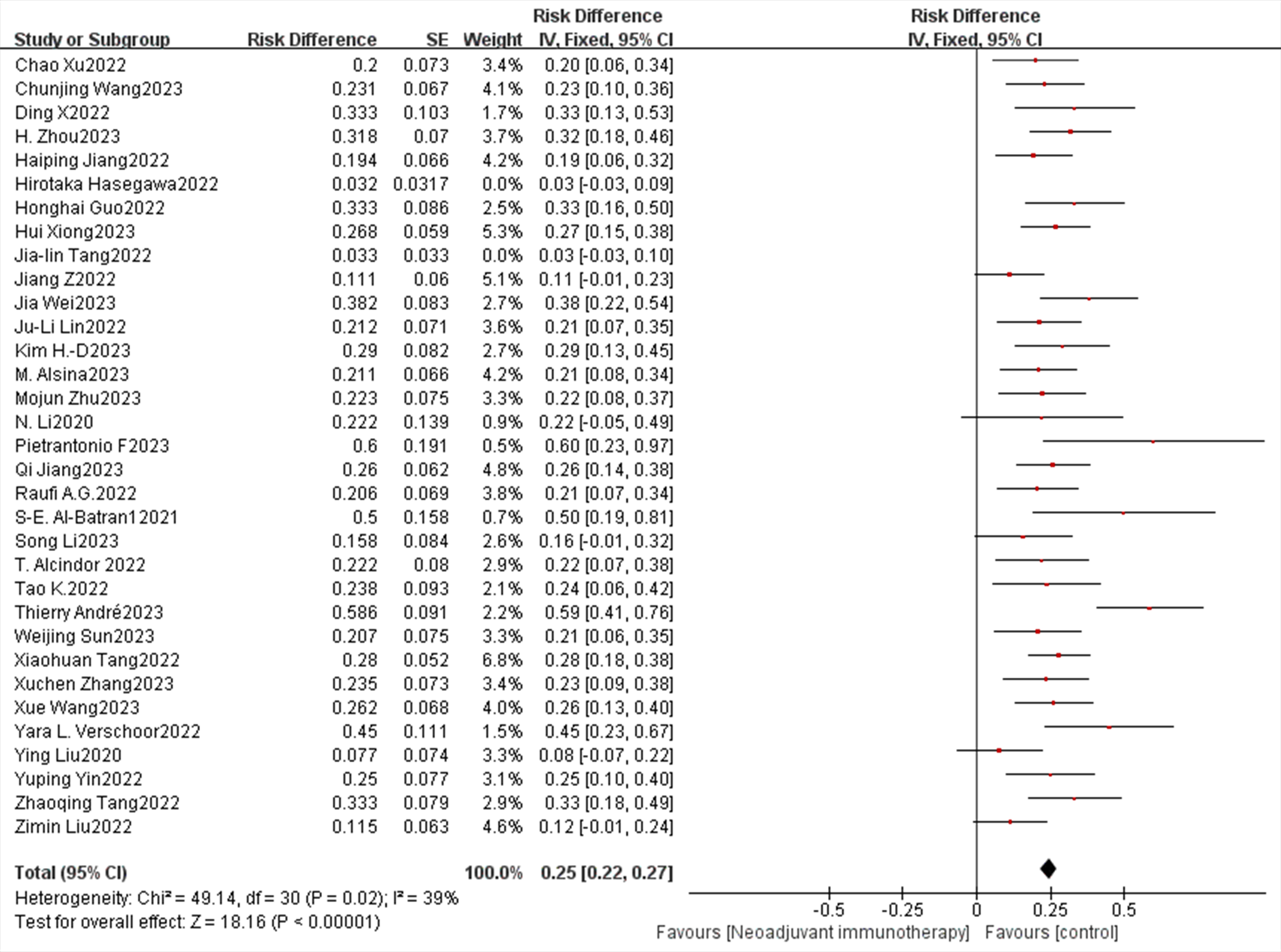

Supplement: Supplementary Figure 3 — Sensitivity analysis of the incidence of PCR. [file Image_3.tif]

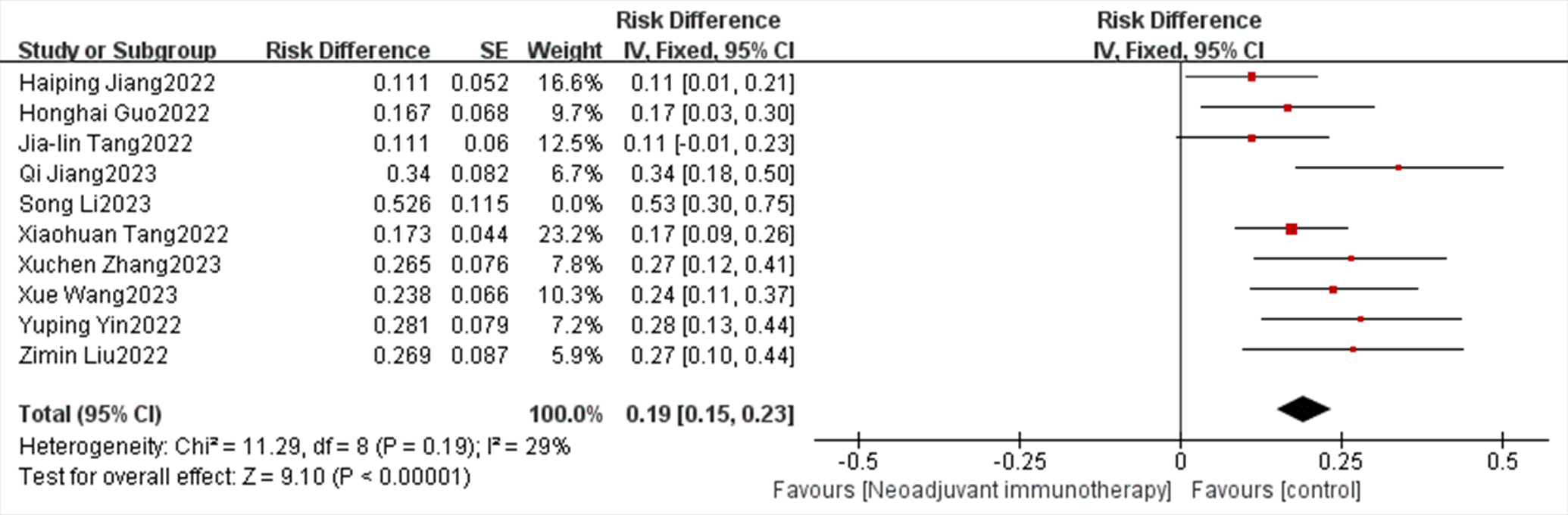

Supplement: Supplementary Figure 4 — Sensitivity analysis of the incidence of TRG3. [file Image_4.tif]

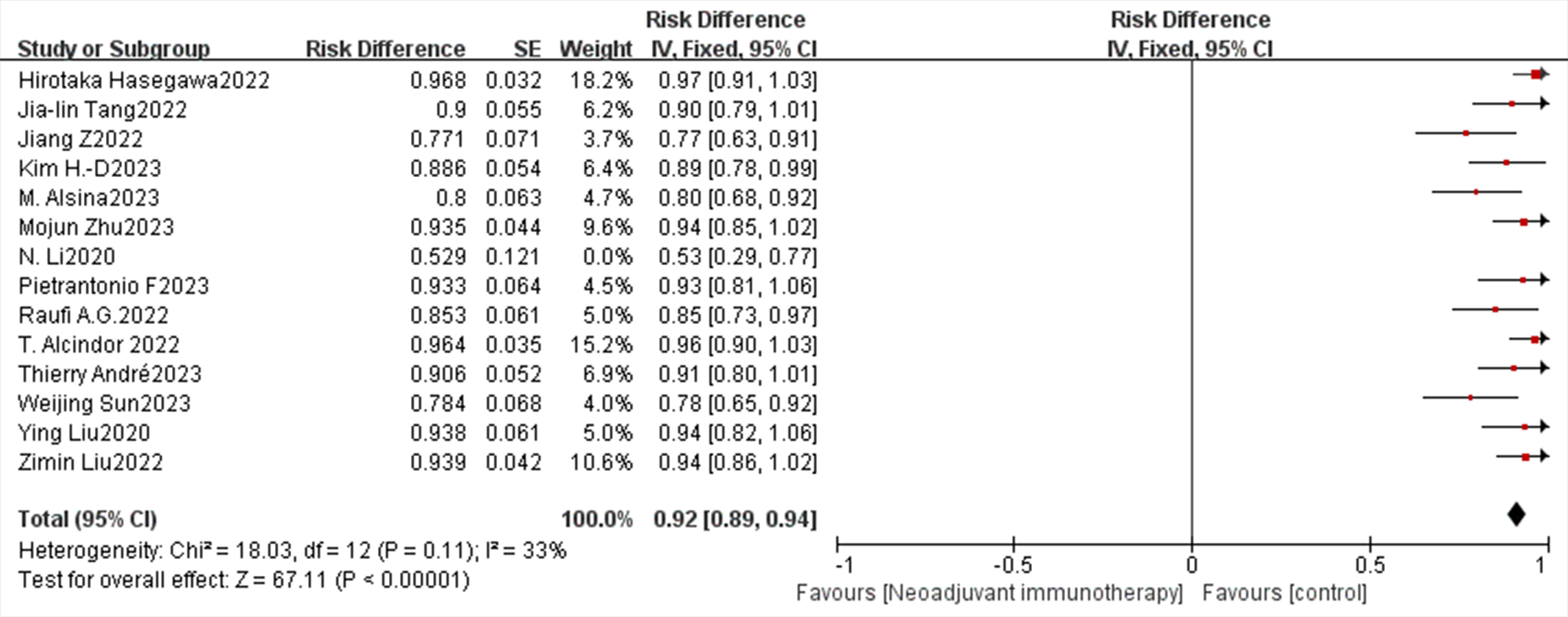

Supplement: Supplementary Figure 5 — Sensitivity analysis of the surgical resection rate. [file Image_5.tif]

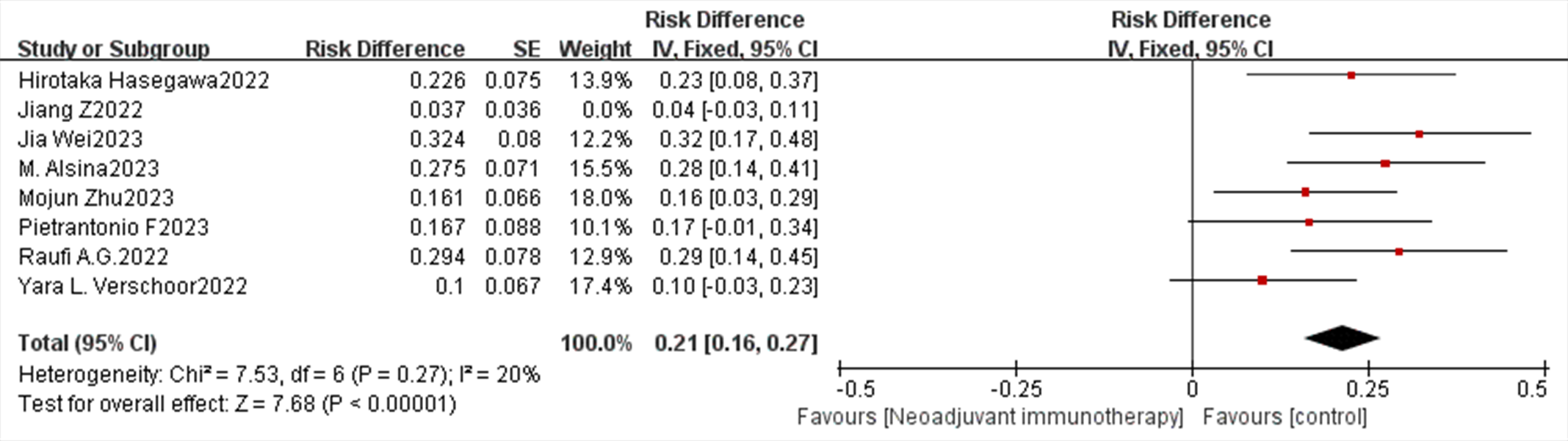

Supplement: Supplementary Figure 6 — Sensitivity analysis of the incidence of ≥ 3irAEs. [file Image_6.tif]

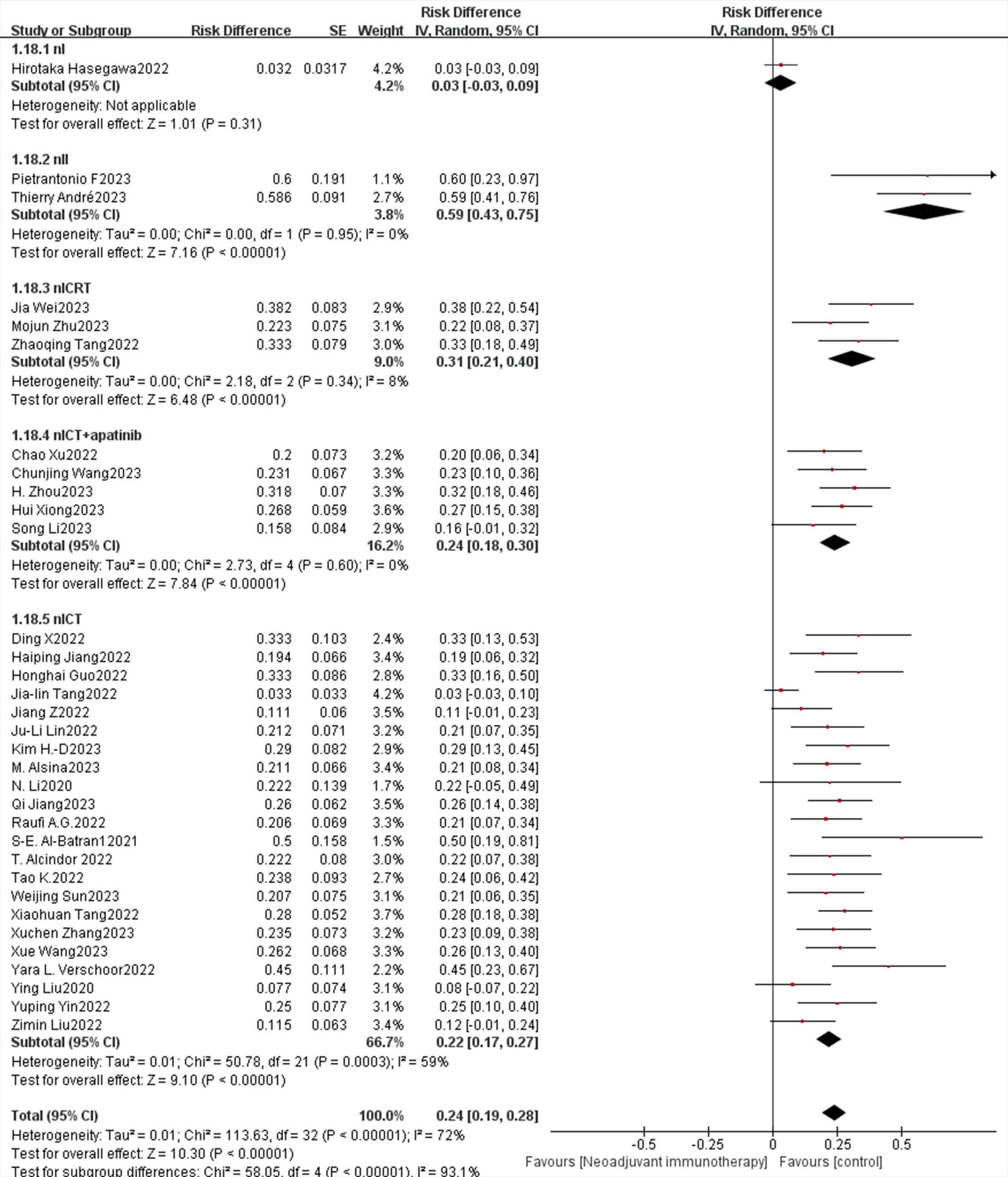

Supplement: Supplementary Figure 7 — Subgroup analysis of PCR. [file Image_7.tif]

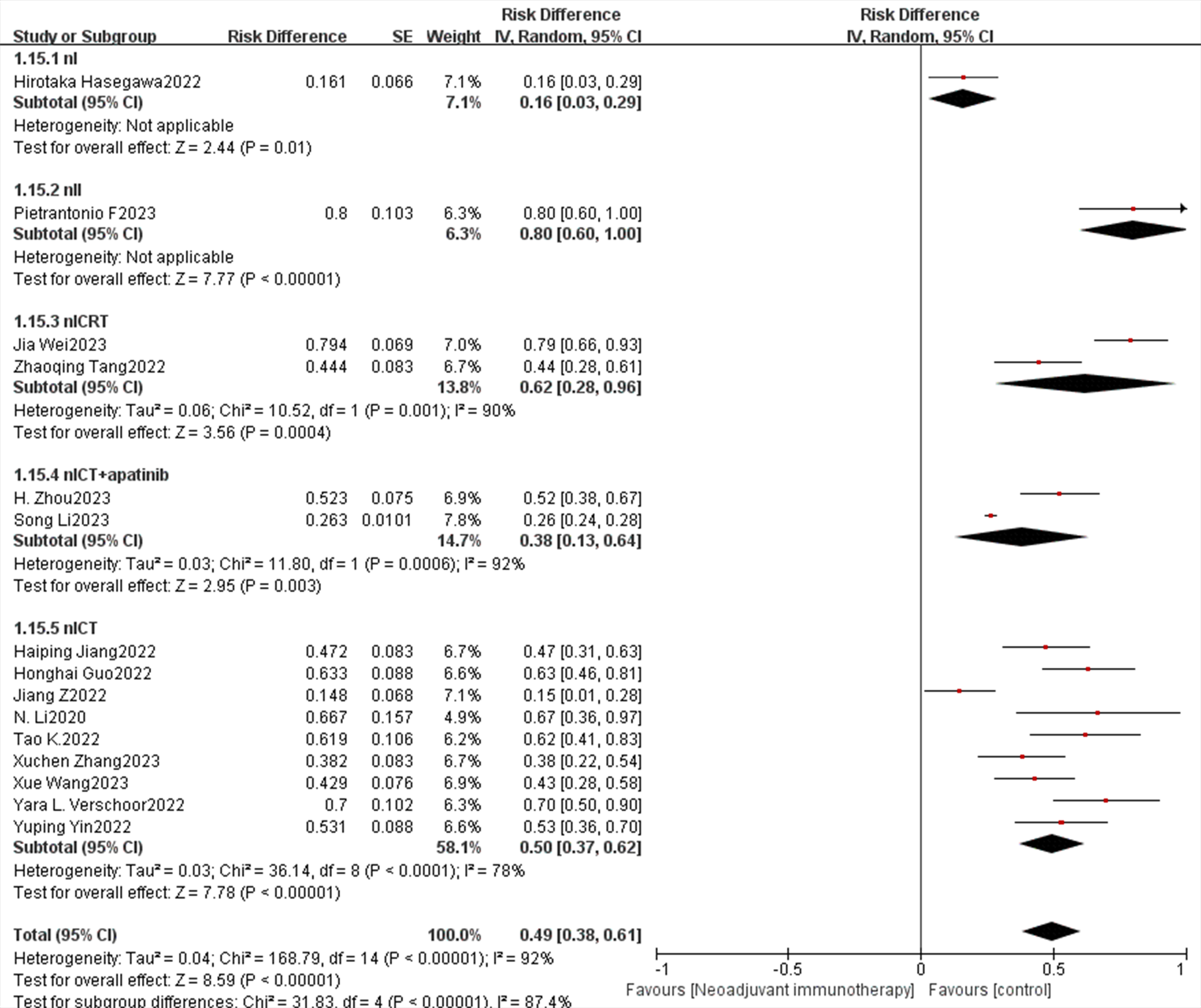

Supplement: Supplementary Figure 8 — Subgroup analysis of MPR. [file Image_8.tif]

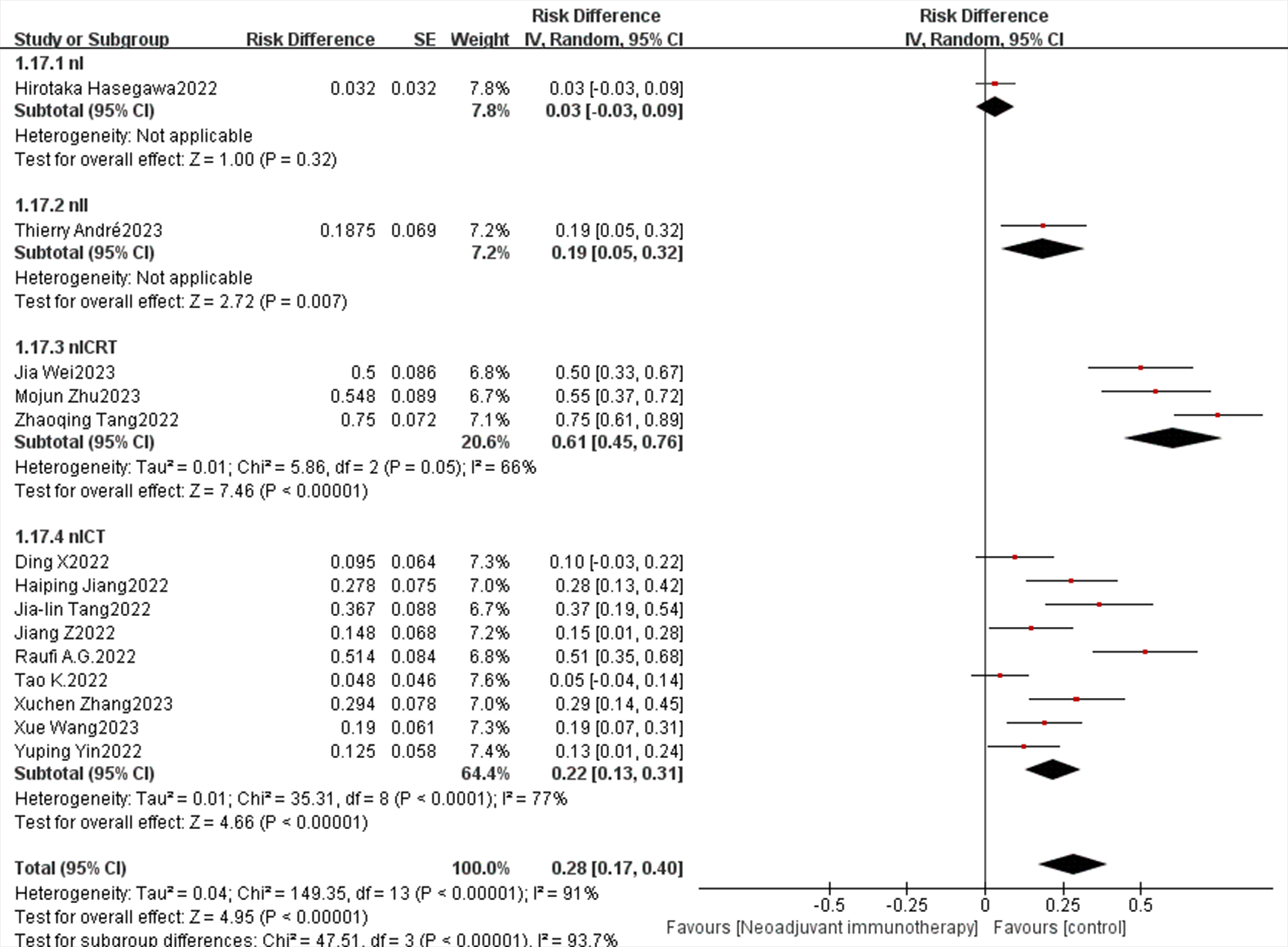

Supplement: Supplementary Figure 9 — Subgroup analysis of the incidence of ≥ 3TRAEs. [file Image_9.tif]
